# Effectiveness of behavioral-cognitive group therapy on improvement of quality of life of patients with coronary heart disease

**Published:** 2015

**Authors:** M Talebi Amri, M Bahraminasab, E Samkhaniyan, F Moini, Z Kazemi Khobane

**Affiliations:** * Clinical Psychology, Islamic Azad University, Roudehen Branch, Iran; ** Educational Management, Islamic Azad University, Garmsar Branch, Iran; *** Health Psychology, Department of Education and Psychology, Islamic Azad University, Karaj, Iran; **** Clinical Psychology, Islamic Azad University, Ayatollah Amoli Branch, Iran; ***** Clinical Psychology, Islamic Azad University, Karaj Branch, Iran

**Keywords:** group therapy, cognitive-behavioral, anxiety, stress, coronary heart disease

## Abstract

**Objective:** An appropriate psychological intervention to promote the level of the psychological health of patients with a coronary heart has a great importance. The principal intention of the current study was to study the efficacy of the behavioral-cognitive group therapy on the quality of life of the cases with a coronary heart illness.

**Method:** The current research was a quasi-test via posttest-pretest that was used by the checking team. Hence, 24 patients with coronary heart disease were selected by using the convenience sampling technique and were placed in experimental and control groups in Shahid Rajaee Heart Center in Tehran. Both groups were pretested by using a demographic questionnaire and a quality of life questionnaire. Afterwards, the experimental group trained for eight sessions of cognitive-behavioral group therapy, and the control group gained no interference. Later, both groups were post-tested, and the acquired information was examined by using inferential and descriptive statistical methods accompanied by SPSS 21 software.

**Findings:** The results indicated that the cognitive-behavioral group therapy training significantly increases the quality of life of cases with coronary heart problems.

**Conclusion:** The conclusions of the existing research were that due to the high level of the effectiveness of the cognitive-behavioral group therapy training, its low cost and high acceptability by the patients, especially when it was performed in a group, it had a significant positive impact on the enhancement of the quality of life in cases of coronary heart illness.

## Introduction

In the 17th century, Harvey (1628) [**15**] stated that “any impact on mind accompanied by pain, satisfaction, pleasure, fear, or hope is associated with heart stimulation”. Believing that one’s psychological states could influence his physical state has a long history. The term “Psychosomatic Disorders” or “Psychophysiological Disorders” refer to the physical states that the significant physiological occurrences in them have a close relationship with physical symptoms [**4**]. The emergence of psychosomatic disorders demands a permanent proximity and transplantation of psychological factors and physical symptoms simultaneously [**7**]. Cardiovascular diseases are significant mental disorders [**12**]. Among heart diseases, the coronary heart disease has a psychosomatic nature and the effect of social and psychological factors (personality factors in particular) is significant in its emergence [**6**]. Some of the researchers believe that social and psychological factors are the most important parameters in the emergence and continuing of the coronary heart problems [**9**]. Moreover, this disease has a high impact on the psychological moods and quality of life of cases via coronary heart problems mutually.

The related researches showed that there was a bilateral and mutual link between health and quality of life [**6**]. The undesirable quality of life accompanied by the worsening of the disease, less possibility of survival, increasing the number of days of hospitalization and decreasing the functional activity in patients with heart disease [**11**]. In other words, individuals who have physical diseases, especially diseases that make them incapable and chronic illnesses such as heart disease, are faced with a variety of problems affecting their daily and routine life and are led to a reduction in the quality of life [**16**].

The health-related quality of life is a subset of the overall quality of life that consisted of the feeling of subjective, emotional, physical, and social well-being and reflects the patients’ mental evaluation and the way of responding to the illness [**11**]. The high prevalence of chronic diseases including coronary artery disease demands needs paying a particular attention to the measurement of the quality of life [**16**]. The psychological states are among the factors affecting the quality of life in cases via chronic physical illnesses, especially heart problems [**7**], the quality of life in such patients being increased by the undergoing of different medical and psychological interventions as complementary therapies.

The behavioral-cognitive therapy is among the therapeutic interventions, whose effectiveness was approved in a variety of purposes. Behavioral-cognitive therapy is a kind of therapy that emphasizes the way of thinking of a person. In other words, emotions and feelings of a person are influenced by his/ her way of thinking and help the person perceive emotions, feelings, and attitudes that affect his/ her behaviors [**17**]. Behavioral-cognitive therapy is the short-term most of the time, the person learning how to identify and change destructive and annoying thought patterns and emotions that have an adverse effect on his/ her behaviors during treatment, which is usually eight to twelve sessions [**3**]. According to the behavioral-cognitive therapy approach, the discussing of concepts operationally and the validation of the treatment experimentally is emphasized. The major part of the treatment is based on the approach of “Here and Now” and it is assumed that the primary purpose is to help the patient create desirable changes in his/ her life [**2**]. Therefore, during the treatment, people learn to control their thoughts and identify the causes of the feelings and actions. Moreover, they are provided opportunities for new adaptive learnings and creating changes in the external environment in clinical scopes [**18**.

According to the above-mentioned information, it seems that the behavioral-cognitive therapy has a great impact on the enhancement of the quality of life in cases suffering from coronary disease; thereby the present investigation studied the efficacy of behavioral-cognitive group therapy on the quality of life quality of the cases.

## Methodology

The current analysis is a quasi-test via posttest-pretest that used a checking group. The population consisted of all the patients with a coronary heart disease, who referred to Shahid Rajaee Health Hospital in Tehran from December to March 2015. According to the fact that the minimum sample population in the experimental studies should be of 15 individuals, a 15-individuals sample size (n = 15) was chosen for each of the groups [**10**]. The inclusion standards of the existing study were a diagnosis of coronary heart disease based on the diagnosis of cardiologists that included the following indexes.

Informed happiness and eagerness to cooperate in the research.

Ability to take part in sessions and cooperation in doing assignments.

Willingness to cooperate in completing instruments.

Physical and psychological stability (no apparent physical or psychological signs that could intervene during the sessions, such as shortness of breath).

The age ranged between 20 and 45. Moreover, the patients would neglect it if there were no possibility to continue the study due to the existence of a physical or psychological illness or existence of any cognitive disorder or impaired cognitive function. This way, some of the patients in Shahid Rajaee Heart Hospital were selected and were placed into two groups of control and cognitive-behavioral therapy accidentally.

The implementation method meant that the sampling of all the patients was conducted in the hospital, when referring to Shahid Rajaee Heart Hospital. Afterwards, 40 individuals were randomly selected and placed in two different groups (20 in each group) after having ensured the inclusion and exclusion criteria. Further, some explanations were provided regarding the treatment sessions and the research questionnaire and presented to the patients. In the case of an individual’s approval to participate in the study, he/ she was randomly placed in one of the groups. Prior to the implementation of the research, in order to observe the ethical principles and to ensure the attendance at meetings, informed consents were obtained in addition to explaining the investigation and its positive impacts; then, the experimental group was trained for eight sessions of cognitive-behavioral group therapy and the control group received no intervention. Finally, both groups were post-tested. The protocols employed for the behavioral-cognitive group therapy sessions were mentioned in **Table 1**.

The instruments used in the present research consisted of the demographic questionnaire and MacNew heart disease health-related quality of life questionnaire (HRQL). 

Demographic survey: this questionnaire was formulated to receive the personal information of participants. Characteristics like gender, age, marital status, and education were questioned in the survey.

MacNew heart disorder health-associated quality of life survey: is a self-report tool for patients with heart disease, which was developed in English by Lim and Oldridge (1994) and was edited by Valenti (1996). This questionnaire is the edited form of the Quality of Life after Myocardial Infarction (QLMI) [**14**]. This instrument was the standardized in the Iranian population in 2013 by Asadi-Lari, Javadi, Melville, and Oldridge [**8**] and consisted of 27 questions. Each subject was classified according to a seven-point Likert rate (always to never) and the average response time was equal to 10 minutes. This questionnaire measured the quality of life from three points of view: emotional (questions number 1-8, 10, 12, 13, 15, 18 and 23), physical (questions number 6, 9, 12, 14, 16, 17, 19, 20, 21, 24-26) and social (questions number 2, 11-13, 15, 17, 20-26) [**8**]. Five questions in the field of physical performance evaluated the symptoms of illness, including symptoms of breath shortness, chest pain, dizziness, leg pain, and fatigue. The distribution of questions was in such a way that each question could be placed in one, two or three purposes. These questions were scored as it follows: the maximum score could be equal to 7, which indicated very good health-associated quality of life and the least score could be equal to 1, which indicated a very weak health-associated quality of life. In addition, this questionnaire presented 4 scores, including the overall score for health-associated quality of life (by calculating the summation of scores for all the questions), a score related to the emotional dimension, a score related to the physical dimension and a score related to the social dimension [**13**]. According to a study conducted by Asadi-Lari et al. (2003) [**8**], the mentioned reliability of the questionnaire in the Iranian population was calculated to be equal to 0.92, 0.92, 0.95, and 0.95 for the emotional, physical, emotional dimensions and the overall score by using Cronbach’s alpha method. Moreover, its correlation coefficient was calculated to be equal to 0.42, 0.38, 0.31, and 0.50 for the emotional, physical, emotional dimensions and the overall score. The internal consistency of the questionnaire was equal to 0.92, 0.92, 0.93, and 0.95 for the emotional, physical, emotional dimensions and the overall score. Moreover, by using construct validity, the reliability of emotional, physical, and social dimensions was in the range of 0.630 to 0.665.

The Statistical Package for Social Sciences (SPSS-21) software was used to analyze the obtained data. The analysis of the research data was based on descriptive statistics, frequency, its percentage, mean indices, and nominal deviation used. Moreover, multi variable Analysis of Covariance (ANCOVA) was used based on inferential statistics.

**Table 1:** Cognitive-behavioral group therapy training protocol

Session	Subject
First	Referral of group members, being familiar with group policy, introduction of depression, anxiety and stress, and being aware of their physical effects
Second	Recognizing negative thoughts, way of creating these thoughts, learning to overcome negative thoughts
Third	Training to overcome dichotomous thinking, training to overcome the arbitrary interpretations, training to overcome unbalanced judgments, training to overcome immediate conclusion, training to overcome mind-reading, wrong impressions
Fourth	Training to overcome extreme generalization, training to overcome labeling, training to overcome inexact term, training to overcome exaggerated generalization, training to overcome absolutism, training to overcome feeling guilty, and training to overcome mental filtering
Fifth	Training to overcome zooming in and out, training to overcome tragic consequences, training not to be disastrous, training to overcome split swiftness, training to overcome too much attention to negative situations and training to overcome personalization
Sixth	Being aware of the time of getting angry, controlling anger and overcoming anger
Seventh	Continuing training, practicing and performing exercises, training for relaxation techniques to use in uncomfortable situations
Eighth	Briefly overviewing the sessions and providing feedback to each other, training to transfer data and findings to the external environment of the group

**Research Findings**

The demographic characteristics of the present sample size is listed in **Table 2**.

**Table 2:** Demographic characteristics of the subjects

Variable	Group	Frequency	Frequency percentage	Mean and standard deviation
Age	25-30	6	15	36.95 ± 6.23
	31-35	12	30	
	36-40	6	15	
	41-45	16	40	
Gender	Male	24	60	
	Female	16	40	
Level of education	High School Diploma	12	30	
	Associate Degree	12.5	15	
	Bachelor degree	19	47.5	
	Master degree	4	10	
Marital status	Bachelor	8	20	
	Married	32	80	

**Table 3:** Descriptive statistics of scores of research variables in the two groups according to the pretest and post test

Component	Index	Experiment		Control	
		Pretest	Posttest	Pretest	Posttest
Physical	Average	34.55	50.95	39.05	39.65
	Standard deviation	7.65	6.90	3.28	3.10
Emotional	Average	44.05	68.65	47.80	48.40
	Standard deviation	7.16	10.83	7.17	6.99
Social	Average	38.75	63.50	38.25	38.50
	Standard deviation	4.42	7.48	4.44	4.49

According to **Table 3**, the average scores of physical, emotional, and social dimensions were increased in the test team related to the checking team.

**Table 4:** Levene test results which investigate the default homogeneity of variances of physical, emotional and social dimensions in posttest

Variable	Stage	F	Df1	Df2	Sig. level
Physical	Posttest	0.418	1	38	0.552
Emotional	Posttest	0.991	1	38	0.326
Social	Posttest	0.569	1	38	0.455

**Table 4** presents the null hypothesis of equality of variances of the two groups in physical, emotional, and social dimensions, which were approved. In other words, the variances of the two groups are equal to each other regarding the physical, psychological, and social dimensions and there is no significant difference. Therefore, according to the compliance with Levine defaults, the results of the research hypotheses are permissible.

**Table 5:** Results of multivariable ANACOVA on the scores of posttest with control of pretest in physical, emotional, and social dimensions

Test	Value	F	Df	Sig. level	Squared Eta	Power
Play effect	0.863	75.853	3	0.001	0.863	0.95
Wilks Lambda	0.137	75.853	3	0.001	0.863	0.95
Hotelling effect	6.321	75.853	3	0.001	0.863	0.95
Ray’s largest root	6.321	75.853	3	0.001	0.863	0.95

As mentioned in **Table 5**, the significance level of all the tests (P < 0.001) revealed that there were variations between the 2 teams in at least one of the related parameters (physical, emotional and social dimensions). According to the squared eta, 0.86 percent of the seen distinctions among persons was linked to the effect of the free parameter (i.e. intervention method). On the other hand, since the statistical power was equal to 0.95 (greater than 0.80), the sample size was admissible. The findings linked to the clear variation of each of the dependent variables were mentioned in the following. 

**Table 6:** Results of multivariable ANACOVA in order to investigate the effectiveness of behavioral-cognitive group therapy training on physical, emotional, and social dimensions in posttest

Index	Sum of squares	Df	Mean Square	F	Sig. level	Squared Eta
Physical	1276.901	1	1276.901	44.618	0.001	0.541
Emotional	4101.625	1	4101.625	49.321	0.001	0.565
Social	6250.001	1	6250.001	164.019	0.001	0.812

Based on **Table 6**, since p < 0.001, the hypothesis related to the differences between the physical, emotional, and social dimensions between the two groups was approved. Moreover, it was expressed that 0.54 percent of the change in the score of physical size, 0.56 percent of change in the score of emotional dimension, and 0.81 percent of change in the score of social dimension is due to independent variable (behavioral-cognitive group therapy training). Therefore, it was expressed that the behavioral-cognitive group therapy training leads to the improvement of physical, emotional, and social dimensions in cases with coronary heart problems.

## Conclusion

According to the present study on the effectiveness of behavioral-cognitive group therapy training on the quality of life in cases with coronary heart problems, the results obtained from multivariable ANACOVA indicated that that behavioral-cognitive group therapy training had a significant impact on the increase of the quality of life in the cases. These results were in agreement with the researches performed by Mozafari (2015) [**5**], Aminian et al. (2014) [**1**], Khodaie et al. (2012) [**2**] and Khayyam-Nekuei et al. (2010) [**3**].

In their research, Khayyam-Nekuei et al. (2010) [**3**] stated that there is a significant relationship between one’s perception of himself, his self-confidence, ability to create a positive feeling and resistance to negative emotions, positive views about himself, his world and his future and realism in coping with chronic illness, especially cardiovascular diseases. If the mentioned factors strengthened in the patients, they could have a great impact on the sense of improvement, satisfaction, experience of fear and anxiety; thereby there could be a promotion in the quality of life.

Khodaie et al. (2012) [**2**] stated while explaining the efficacy of behavioral-cognitive therapy on reducing the unpleasant psychological moods such as depression in cases with coronary heart problems, that the existence of a critical component such as the behavioral activation together with the cognitive treatments results in the reduction of depression initially and the decrease in the depressed mood in the case of continuing such treatments. Therefore, it can be deduced that paying attention to negative attitudes and emotions leads to a depressed mood. Using active and productive behaviors, dysfunctional cognitive changes and increase in self-efficacy take place. As a result, a depressed mood changes to balanced mood and quality of life increases.

**Acknowledgement**

The authors would like to thank the respected authorities of Shahid Rajaee Heart Center for their assistance. Moreover, they would like to thank all the participants for their cooperation in the present research. 
